# Association of kidney function with inflammatory and procoagulant markers in a diverse cohort: A cross-sectional analysis from the Multi-Ethnic Study of Atherosclerosis (MESA)

**DOI:** 10.1186/1471-2369-9-9

**Published:** 2008-08-05

**Authors:** Christopher Keller, Ronit Katz, Mary Cushman, Linda F Fried, Michael Shlipak

**Affiliations:** 1 Department of Medicine, University of California San Francisco, San Francisco, CA, USA; 2 San Francisco Veterans Affairs Medical Center, General Internal Medicine Section, San Francisco, CA, USA; 3 Collaborative Health Studies Coordinating Center, University of Washington, Seattle, WA, USA; 4 Departments of Medicine and Pathology, University of Vermont, Burlington, VT, USA; 5 Renal Section, Veterans Affairs Pittsburgh Healthcare System, Pittsburgh, PA, USA

## Abstract

**Background:**

Prior studies using creatinine-based estimated glomerular filtration rate (eGFR) have found limited associations between kidney function and markers of inflammation. Using eGFR and cystatin C, a novel marker of kidney function, the authors investigated the association of kidney function with multiple biomarkers in a diverse cohort.

**Methods:**

The Multi-Ethnic Study of Atherosclerosis consists of 6,814 participants of white, African-American, Hispanic, and Chinese descent, enrolled from 2000–2002 from six U.S. communities. Measurements at the enrollment visit included serum creatinine, cystatin C, and six inflammatory and procoagulant biomarkers. Creatinine-based eGFR was estimated using the four-variable Modification of Diet in Renal Disease equation, and chronic kidney disease was defined by an eGFR < 60 mL/min/1.73 m^2^.

**Results:**

Adjusted partial correlations between cystatin C and all biomarkers were statistically significant: C-reactive protein (r = 0.08), interleukin-6 (r = 0.16), tumor necrosis factor-α soluble receptor 1 (TNF-αR1; r = 0.75), intercellular adhesion molecule-1 (r = 0.21), fibrinogen (r = 0.14), and factor VIII (r = 0.11; two-sided p < 0.01 for all). In participants without chronic kidney disease, higher creatinine-based eGFR was associated only with higher TNF-αR1 levels.

**Conclusion:**

In a cohort characterized by ethnic diversity, cystatin C was directly associated with multiple procoagulant and inflammatory markers. Creatinine-based eGFR had similar associations with these biomarkers among subjects with chronic kidney disease.

## Background

Higher levels of markers of inflammation, such as C-reactive protein (CRP) and interleukin 6 (IL-6), have been associated with cardiovascular disease in healthy populations [1-3]. In subjects with end stage renal disease (ESRD), inflammatory biomarkers are significantly elevated and predict poor outcomes [4-7]. In subjects with kidney disease not on hemodialysis, kidney function has been associated with markers of inflammation for creatinine-based estimated glomerular filtration rates (eGFR) below 60 mL/min/1.73 m^2^. Above that threshold, two studies did not find an association between eGFR and markers of inflammation [8,9].

Cystatin C, a cysteine protease inhibitor secreted by all nucleated cells, is a novel serum marker for kidney disease that may better detect small changes in kidney function [10-12]. Since creatinine-based eGFR is not reliable above 60 mL/min/1.73 m^2^, cystatin C may be superior in detecting an association with inflammation in subjects with mild to moderate kidney disease [13,14]. Using cystatin C as a marker for kidney function in an ambulatory elderly cohort, all with creatinine-based eGFR ≥ 60, we reported linear associations between cystatin C and five inflammatory markers: CRP, IL-6, tumor necrosis factor alpha (TNF-α), and two soluble TNF-α receptors [15]. In a cohort with known coronary artery disease, cystatin C was associated with both CRP and fibrinogen across the entire cohort, while creatinine-based eGFR was significantly associated with CRP and fibrinogen only for eGFR < 60 [16].

The current study investigated the association of both creatinine-based eGFR and cystatin C with six inflammatory and procoagulant biomarkers in the Multi-Ethnic Study of Atherosclerosis (MESA), a large cohort characterized by racial and ethnic diversity. Compared with prior studies on kidney function and markers of inflammation, this study featured a population with four racial/ethnic groups, a younger mean age (62 years), no clinical cardiovascular disease, and more extensive measurements of both inflammatory and procoagulant biomarkers. In addition, a second objective of this study was to test whether the association between kidney function and multiple inflammatory and procoagulant biomarkers differed by race/ethnicity.

## Methods

The MESA cohort consists of 6,814 men and women who identified themselves as white, African-American, Hispanic, or Asian (of Chinese descent). At the time of enrollment, the subjects were 45 to 84 years of age and free of clinical cardiovascular disease (CVD). Participants were enrolled from July 2000 to August 2002, and recruited from six US communities. Subjects were initially recruited using residential and telephone listings; towards the end of the recruitment period, lists of Medicare beneficiaries and participant referrals were also used to ensure an adequate number of participants. The study was approved by institutional review boards at each center, and all study participants gave informed consent.

All biochemistry assays were performed on plasma or serum drawn in the morning after an overnight fast during the initial visit and stored at -70°C. Cystatin C was measured using a BNII nephelometer on plasma specimens (N Latex Cystatin C; Dade Behring Inc., Deerfield, IL) [17]. The assay range is 0.195 to 7.330 mg/L, with the reference range for young, healthy individuals reported as 0.53 – 0.95 mg/L. Intra-assay coefficients of variation (CVs) range from 2.0 – 2.8% and inter-assay CVs range from 2.3 – 3.1%. Serum creatinine was measured using colorimetry with a Johnson & Johnson Vitros 950 analyzer (Johnson & Johnson Clinical Diagnostics Inc., Rochester, NY). The CVs for serum creatinine were ≤ 2%. Estimated GFR was calculated using the creatinine-based four-variable Modification of Diet in Renal Disease (MDRD) equation, which features adjustments for age, gender, serum creatinine, and black race [18]. Creatinine levels were calibrated to the Cleveland Clinic standard (0.9954*Cr + 0.0208) [19]. Lipid measurements were performed using the Roche COBAS FARA centrifugal analyzer (Roche Diagnostics, Indianapolis, IN). Low-density lipoprotein cholesterol (LDL) was calculated using the Friedewald equation [20]. Serum glucose was measured using the Vitros 950 analyzer. Urine samples for measuring creatinine and albumin were taken from a spot urine collection at the initial visit. Urinary creatinine was measured using the Vitros 950. Urinary albumin was measured using an Array 360 CE Protein Analyzer (Beckman Instruments Inc., Fullerton, CA). Chronic kidney disease was defined as eGFR < 60 mL/min/1.73 m^2^[13].

CRP was measured using a BNII nephelometer (N high sensitivity CRP; Dade Behring Inc.), with intra-assay CVs of 2.3% – 4.4%, inter-assay CVs of 2.1% – 5.7%, and a detection level of 0.18 mg/L [21]. IL-6 was measured by ultra-sensitive ELISA (Quantikine HS Human IL-6 Immunoassay; R&D Systems, Minneapolis, MN) with an analytical CV of 6.3% and a detection level of 0.04 pg/mL. Tumor necrosis factor alpha receptor 1 (TNF-αR1) was measured by ultra-sensitive ELISA (Quantikine Human sTNF RI Immunoassay; R&D Systems) with an analytical CV of 5.0% and a detection level of 0.77 pg/mL. Intercellular adhesion molecule 1 (ICAM-1) was measured by ELISA (Parameter Human sICAM-1 Immunoassay; R&D Systems), with a CV of 5.0%. Fibrinogen was measured using a BNII nephelometer (N Antiserum to Human Fibrinogen; Dade Behring Inc.) with intra-assay and inter-assay analytical CVs of 2.7% and 2.6%, respectively. Factor VIII coagulant activity was determined by measuring the clotting time of a sample in factor VIII deficient plasma in the presence of activators utilizing the Sta-R analyzer (STA-Deficient VIII; Diagnostica Stago, Parsippany, NJ), with a reported normal plasma range in the adult population of 60–150%. CRP, IL-6, fibrinogen, and factor VIII were measured in the entire cohort. Intercellular adhesion molecule-1 (ICAM-1) was measured in a total of 2,614 subjects, including all participants who enrolled before February 2003 and a subset of 1,000 participants randomly selected from the first 5,030 participants enrolled. The baseline characteristics of the random subset of participants were not significantly different from those of the entire MESA cohort. TNF-αR1 (995 samples) was measured in a subset of the cohort chosen randomly after 75% of the participants had been enrolled.

Participant characteristics, including demographics (age, sex, race/ethnicity); comorbid conditions (diabetes, defined as a fasting glucose ≥ 126 mg/dL (6.9 mmol/L) or by the use of insulin or oral hypoglycemic medications, and hypertension, defined as an average systolic blood pressure ≥ 140 mm Hg, an average diastolic blood pressure of ≥ 90 mm Hg, or by the use of antihypertensive medications), smoking history, defined as ever [current or former] or never; and statin use were obtained at the enrollment visit using data from standardized questionnaires. Resting blood pressure was determined by taking three measurements with the participant in the seated position; systolic and diastolic blood pressures were recorded as the average value of the last two measurements from both the first and second study examinations. Body mass index (BMI) was calculated at the initial visit using weight (kg) divided by height squared (m^2^).

### Statistical Analysis

Baseline characteristics were evaluated for statistical significance across quintiles of cystatin C using ANOVA or chi-square tests for trend. Histograms and q-q plots revealed that the urinary albumin to creatinine ratio and the levels of CRP and IL-6 were skewed; therefore, logarithmic transformations of these variables were used for analysis. First, partial correlations with each of the six biomarkers were determined separately for cystatin C and creatinine-based eGFR, adjusting for age, gender, race/ethnicity, and BMI. Two-tailed t-tests were used for significance testing of all partial correlations. We also compared the partial correlation coefficients of eGFR and cystatin C with each biomarker after stratification by presence or absence of CKD. The two correlation coefficients were transformed using the Fisher Z-transform and the difference and a p-value were computed.

We created separate linear regression models for each of the six biomarkers. The primary predictors were either cystatin C (per SD) or eGFR by the four-variable MDRD equation, dichotomized as < and ≥ 60 ml/min/1.73 m^2^. Covariates from Table 1 were entered into the models based on their potential role as confounders due to their associations with both kidney disease and inflammation. We also determined the adjusted mean levels of each biomarker, stratified by the presence of CKD (eGFR < 60 mL/min/1.73 m^2^) and by quintile of cystatin C. The mean biomarker levels were also plotted across quintiles of cystatin C, with the y-axis scale standardized at ± 1 SD of the overall mean of the cohort for each biomarker. For TNF-αR1, the y-axis scale was set at ± 2 SD given the larger differences for that biomarker across quintiles of cystatin C. T-tests were used to evaluate significant differences in mean levels in subjects with and without CKD, and ANOVA across quintiles of cystatin C. We also tested for interactions of race/ethnicity with both cystatin C in each regression model. S-Plus (release 6.1, Insightful Inc, Seattle, WA) and SPSS statistical software (release 13.0.1, SPSS Inc, Chicago, IL) were used for the analyses. P < 0.01 was used for statistical significance because multiple comparisons were made and because a high level of power was available.

**Table 1:** Baseline characteristics of MESA population by quintiles of cystatin C

	Cystatin C (mg/L)					
		
	Quintile 1	Quintile 2	Quintile 3	Quintile 4	Quintile 5	
	≤ 0.74	0.75–0.82	0.83–0.89	0.91–1.02	≥ 1.03	
	(n = 1446)	(n = 1377)	(n = 1283)	(n = 1359)	(n = 1285)	
	Mean ± SD, Median [IQR], or %					p-value for trend

Age	57 ± 9	59 ± 9	62 ± 9	65 ± 10	69 ± 10	<0.001
Female, %	65	53	49	47	49	<0.001
Race/Ethnicity, %						
White	33	34	41	41	44	
Chinese	17	13	10	11	8	
African-American	30	30	26	24	28	
Hispanic	19	24	23	24	20	
Smoking History, %	44	49	50	53	53	<0.001
BMI (kg/m^2^)	27 ± 5	28 ± 5	28 ± 5	29 ± 6	30 ± 6	<0.001
Diabetes, %	14	13	12	12	21	<0.001
Hypertension, %	34	36	43	50	64	<0.001
LDL (mg/dL)	117 ± 32	119 ± 31	118 ± 31	117 ± 32	114 ± 32	0.006
HDL (mg/dL)	56 ± 16	51 ± 15	51 ± 14	49 ± 14	48 ± 14	<0.001
Glucose (mg/dL)	105 ± 37	104 ± 32	102 ± 22	104 ± 28	107 ± 31	0.128
Medication use, %						
ACE-inhibitors	0.6	0.9	0.9	1.5	1.9	0.005
Beta-blockers	0.3	0.4	0.9	0.7	0.5	0.427
Statin use	11	14	15	16	19	<0.001
Urinary albumin/creatinine ratio (mg/g)	5.1 [3.4, 10.3]	5.1 [3.2, 9.5]	5.1 [3.1, 9.0]	5.3 [3.3, 10.7]	7.4 [3.9, 20.0]	<0.001
Serum cystatin C (mg/L)	0.68 ± 0.06	0.77 ± 0.02	0.86 ± 0.02	0.96 ± 0.03	1.22 ± 0.36	<0.001
Creatinine-based eGFR (ml/min/1.73 m^2^)	93 ± 16	87 ± 15	82 ± 19	77 ± 13	65 ± 16	<0.001

To convert LDL or HDL from mg/dL to mmol/L, divide by 39; to convert glucose, divide by 18; to convert urinary albumin/creatinine ratio from mg/g to mg/mmol, multiply by 0.114; to convert serum creatinine from mg/dL to μmol/L, multiply by 88.

## Results

The participants of the MESA study had an average age of 62 years. In the cohort, 53% of the participants were female; 39% were white, 28% African-American, 22% Hispanic, and 12% Chinese. The mean cystatin C level in the cohort was 0.89 ± 0.24 mg/L, and the mean creatinine-based eGFR was 81 ± 18 mL/min/1.73 m^2^. On average, the participants in the highest quintile of cystatin C were older, and more likely white and male (Table 1). Those in the highest quintile were also more likely to smoke, to have hypertension and diabetes, to have a higher BMI, to have a higher urine albumin to creatinine ratio, and to have lower levels of both LDL and HDL. Overall, the total number of subjects with CKD in the cohort was 672 (10%).

After adjustment for age, sex, ethnicity, and BMI, cystatin C had statistically significant partial correlations with all six biomarkers in participants both with and without CKD (Table 2, p < 0.01 for all). However, the associations with TNF-αR1 and fibrinogen were significantly stronger among participants with CKD. Similarly, creatinine-based eGFR had significant correlations with all biomarkers except ICAM-1 in subjects with CKD (p < 0.01). In subjects without CKD, eGFR was associated only with TNF-αR1. Of note, the strongest correlations for both cystatin C and creatinine-based eGFR were with TNF-αR1.

**Table 2:** Partial correlations of cystatin C and creatinine-based eGFR with inflammatory and procoagulant markers at the baseline visit of the MESA study, stratified by presence of chronic kidney disease

Marker of Kidney Function	Inflammatory Marker					
	
	CRP (N = 6750)	IL-6 (N = 6622)	TNF-αR1 (N = 995)	ICAM-1 (N = 2611)	Fibrinogen (N = 6750)	Factor VIII (N = 6750)
Cystatin C	0.075*	0.156*	0.748*	0.209*	0.137*	0.112*

Creatinine-based eGFR	-0.019	-0.003	-0.285*	0.025	-0.059*	-0.034*

Persons with CKD						
Cystatin C	0.116*	0.230*	0.903*	0.160*	0.248*	0.140*
Creatinine-based eGFR	-0.101*	-0.139*	-0.763*	-0.035	-0.286*	-0.146*

Persons without CKD						
Cystatin C	0.085*	0.180*	0.557*†	0.261*	0.111*†	0.085*
Creatinine-based eGFR	-0.004‡	0.025‡	-0.168*‡	0.027	-0.021‡	0.017‡

Partial correlations adjusted for age, gender, race/ethnicity, and BMI

Chronic kidney disease (CKD) defined as eGFR < 60 mL/min/1.73 m^2^

* Correlation is significant at the 0.01 level (2-tailed).

† Correlation between cystatin C and the biomarker is significantly stronger among participants with CKD

‡ Correlation between creatinine-based eGFR and the biomarker is significantly stronger among participants with CKD

**Figure 1:**
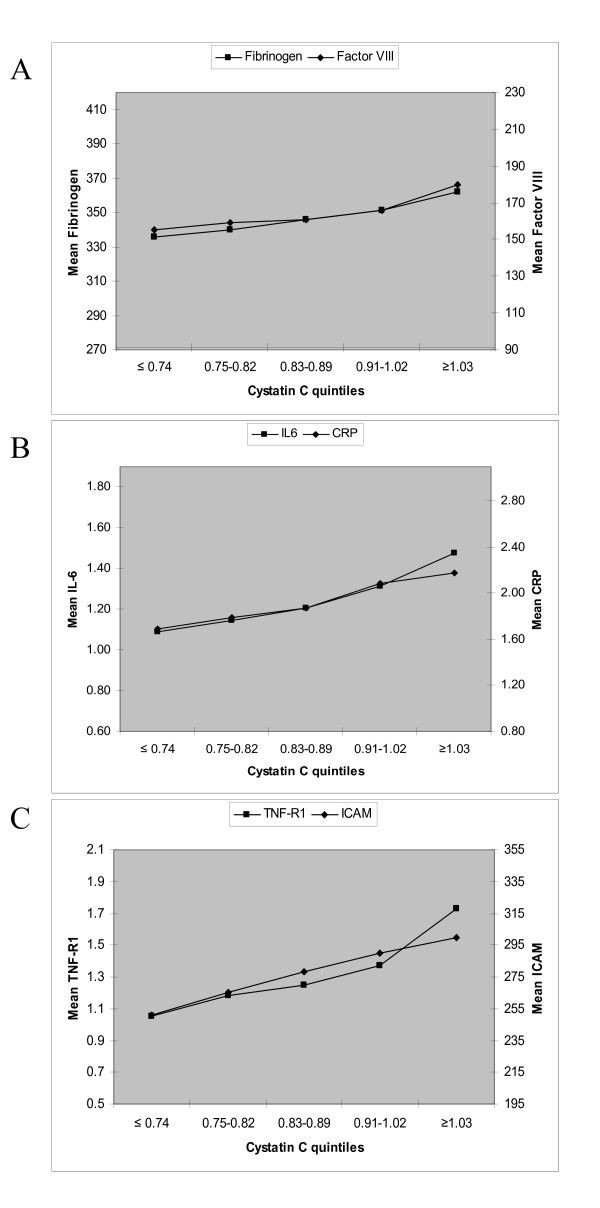
**Adjusted mean biomarkers by quintiles of cystatin C at the baseline visit of the MESA study**. Models adjusted for age, gender, race/ethnicity, smoking status, body mass index, diabetes, hypertension, low-density lipoprotein, high-density lipoprotein, use of statins, and log transformed urinary albumin to creatinine ratio. Y-axis scales are standardized to ± 2 standard deviations of the overall mean of the cohort for TNF-αR1 and ± 1 standard deviation of the overall mean of the cohort for all other biomarkers.

Adjusted mean levels of all biomarkers except ICAM-1 were significantly higher in participants with CKD (eGFR < 60) than in those without CKD (Table 3; p < 0.001 for all). The mean levels of each biomarker in subjects without CKD were unchanged by adjustment for albumin to creatinine ratio. Adjusted mean levels for all six biomarkers increased significantly across the five quintiles of cystatin C (Figures 1a–c). The p-values for trend of the mean biomarker levels across all quintiles were less than 0.01. For comparison, we found that the mean biomarker levels among persons with CKD were similar to the highest quintile of cystatin C in the entire cohort. Finally, adjusted linear regression models using cystatin C to predict biomarker levels were also examined for interactions by race/ethnicity. Statistically significant race/ethnicity interactions were found for the associations of cystatin C with CRP, IL-6, ICAM-1, and factor VIII (Table 4).

**Table 3:** Adjusted mean biomarker levels at the baseline visit of the MESA study, in participants with and without chronic kidney disease

Biomarker (units)	CKD Mean levels (95% CI)	No CKD Mean levels (95% CI)	P-value
CRP (log mg/L) N = 6750	0.81 (0.73, 0.89)	0.63 (0.61, 0.66)	<0.001
IL-6 (log pg/mL) N = 6622	0.37 (0.33, 0.41)	0.19 (0.18, 0.21)	<0.001
TNF-αR1 (pg/mL) N = 995	1.90 (1.76, 2.03)	1.23 (1.22, 1.25)	<0.001
ICAM-1 (ng/mL) N = 2611	281 (271, 290)	274 (271, 277)	0.19
Fibrinogen (mg/dL) N = 6750	363 (360, 372)	345 (343, 346)	<0.001
Factor VIII (%) N = 6750	187 (181, 193)	161 (160, 163)	<0.001

All biomarker models adjusted for age, gender, race/ethnicity, smoking status, BMI, diabetes, hypertension, LDL, HDL, and use of statins

Chronic kidney disease (CKD) defined as eGFR < 60 mL/min/1.73 m^2^

**Table 4:** Associations of cystatin C and biomarker levels at the baseline visit of the MESA study using adjusted linear regression, stratified by race/ethnicity

	White β coefficient (95% CI)	African-American β coefficient (95% CI)	Chinese β coefficient (95% CI)	Hispanic β coefficient (95% CI)	p-value for interaction
CRP (log mg/L)	0.61 (0.37, 0.84)	0.34 (0.14, 0.53)	-0.12 (-0.52, 0.28)	0.13 (-0.06, 0.31)	<0.001
IL-6 (log pg/mL)	0.62 (0.49, 0.75)	0.36 (0.25, 0.47)	0.26 (0.01, 0.51)	0.26 (0.15, 0.36)	<0.001
ICAM-1 (ng/mL)	100.4 (78.8, 122.0)	66.2 (18.7, 113.7)	30.8 (-9.9, 71.6)	48.6 (11.0, 86.3)	0.015
Factor VIII (%)	52.0 (38.3, 65.9)	46.8 (23.5, 70.0)	22.4 (9.0, 35.8)	32.5 (21.3, 43.8)	0.003

All biomarker models adjusted for age, gender, race/ethnicity, smoking status, BMI, diabetes, hypertension, LDL, HDL, and use of statins.

Beta coefficients are given per standard deviation of cystatin C

## Discussion

Our analysis demonstrated that cystatin C was significantly correlated with all six procoagulant and inflammatory biomarkers across a broad range of kidney function, even after adjustment for age, gender, race/ethnicity, and BMI. While creatinine-based eGFR had significant correlations with all biomarkers except ICAM-1 in subjects with CKD, it was only associated with TNF-αR1 in participants without CKD. Adjusted mean levels of all biomarkers increased significantly across each quintile of cystatin C, and all biomarkers except ICAM-1 were elevated in persons with eGFR < 60 compared with eGFR ≥ 60. In general, TNF-αR1 had the strongest correlations with both cystatin C and eGFR in all groups.

Both creatinine-based eGFR and cystatin C correlated with most inflammatory markers in subjects with chronic kidney disease. However, in patients without CKD, only cystatin C had significant correlations with all markers of inflammation. There are several possible explanations for the association of cystatin C with procoagulant and inflammatory biomarkers in patients without chronic kidney disease. One possibility is that GFR is linearly associated with inflammation, and using cystatin C, a marker of renal function that is less dependent on muscle mass or age, reveals the true association between kidney function and markers of inflammation when GFR is greater than 60 mL/min/1.73 m^2^[22]. Prior studies have shown that inflammatory markers are not associated with creatinine-based eGFR above 60 [8,9,16]. However, this absence of association may be due to imprecision of eGFR in the normal range. A second explanation is that cystatin C is associated with inflammation independent of kidney function [23,24]. One study found that cystatin C was associated with CRP independent of creatinine clearance; however, that study did not have the gold standard of measured GFR [25]. Another study, also without a gold standard for GFR, found that the association between cystatin C and CRP disappeared after adjustment for 24-hour urine creatinine clearance [16]. Our observation in this current study, that creatinine-based eGFR and cystatin C have similar associations with inflammatory markers among persons with CKD, makes it seem less likely that cystatin C has a direct association with inflammation that is independent of kidney function. However, our study also lacks a gold standard measurement of GFR, and therefore cannot be conclusive.

Both cystatin C and eGFR had substantial correlations with TNF-αR1, while the associations with the other inflammatory and procoagulant markers were more modest. In a murine model, one study demonstrated that ^125^I-labeled soluble TNF receptors were primarily cleared by the mouse kidney [26]. In contrast, other inflammatory markers, such as CRP and IL-6, are primarily cleared by the liver [27,28]. While the strong association between kidney function and TNF-αR1 levels may be simply attributable to renal clearance of TNF-αR1, TNF-α itself may also play a more complex role in the mediation of kidney damage. A future study should evaluate whether TNF-α and its soluble receptors predict the longitudinal progression of kidney disease.

A secondary goal of our analysis was to assess the role of race/ethnicity in the association between cystatin C and markers of inflammation. The association of four biomarkers – CRP, IL-6, ICAM-1, and factor VIII – with cystatin C had statistically significant interactions by race/ethnicity. Specifically, these four biomarkers had higher beta coefficients in whites compared with other races/ethnicities. One important issue is that, according to recent data, the assay used in MESA is only able to detect certain polymorphisms of ICAM-1 [29]. One allele in particular, ICAM-1 RS5491-T, is more common in African-Americans and not detected by the MESA assay. Further research is ongoing to evaluate the importance of polymorphisms on detection of circulating markers of inflammation. In the MESA study, we are not aware of other assay issues that would affect the interpretation of race/ethnicity interactions with biomarkers.

One possible explanation for the interactions by race/ethnicity in cystatin C models is that regulation of inflammatory cytokines is more dependent on intact kidney function in whites than in other races/ethnicities. Prior studies have shown that CRP and fibrinogen levels are lower in whites than in African-Americans, and that whites at a given baseline level of CRP seem to have slower rises in serum creatinine over time compared with African-Americans [30-32]. These findings would suggest that whites may have a propensity for greater renal excretion of cytokines, although additional studies using urine measurements of cytokines may be helpful to evaluate these associations more effectively. Another explanation for these findings is that cystatin C is a better marker of GFR in whites versus other races/ethnicities. Overall, the association of cystatin C with GFR in non-white groups has not been well studied.

Our analysis has several limitations. First, while cystatin C has been conclusively demonstrated to be a reliable marker of kidney function, it may have associations with inflammation that are dependent of kidney function. Such associations would not be supported by this study, however, since we found that cystatin C and creatinine-based eGFR had equally strong associations with inflammatory markers for subjects when eGFR < 60. Second, as a cross-sectional study, we are unable to determine temporality in the association between cystatin C and multiple biomarkers. For example, inflammation may lead to declining kidney function, or reduced kidney function may lead to elevated inflammatory biomarkers. As stated above, we did not have a gold standard measurement for kidney function, such as iothalamate clearance. We also assumed that eGFR < 60 was the appropriate cutpoint for chronic kidney disease for all subjects, although some data suggests that the established MDRD equation may need to be modified to more accurately characterize CKD race/ethnicity groups other than whites and African-Americans [33].

## Conclusion

We report significant associations between kidney dysfunction and markers of inflammation and procoagulation in a diverse population. Using cystatin C, associations were present in those with and without chronic kidney disease. Creatinine-based eGFR was similarly associated with these biomarkers primarily among subjects with CKD. These results suggest that markers of inflammation are progressively elevated as kidney function declines, even in subjects without chronic kidney disease.

## List of abbreviations

ANOVA: analysis of variance; BMI: body mass index; CKD: chronic kidney disease; CRP: C-reactive protein; CV: coefficients of variation; CVD: cardiovascular disease; ELISA: enzyme-linked immunosorbent assay; ESRD: end stage renal disease; eGFR: creatinine-based estimated glomerular filtration rate; GFR: glomerular filtration rate; ICAM-1: intercellular adhesion molecule-1; IL-6: interleukin-6; LDL: low-density lipoprotein; MDRD: Modification of Diet in Renal Disease; MESA: Multi-Ethnic Study of Atherosclerosis; SD: standard deviation; TNF-α: tumor necrosis factor alpha; TNF-αR1: tumor necrosis factor alpha receptor 1.

## Competing interests

The authors declare that they have no competing interests.

## Authors' contributions

CK was the primary manuscript author and participated in the analysis design and overall data analysis. RK was the primary data analyst and also participated in analysis design and manuscript revision. MC participated in analysis design and manuscript revision. LFF participated in analysis design and manuscript revision. MS participated in the analysis design, overall data analysis, and manuscript revision. All authors have read and approved the final manuscript.

## Pre-publication history

The pre-publication history for this paper can be accessed here:


